# The ‘snowball effect’: short and long-term consequences of early career alcohol industry research funding

**DOI:** 10.1080/16066359.2021.1952190

**Published:** 2021-07-22

**Authors:** Gemma Mitchell, Jim McCambridge

**Affiliations:** Department of Health Sciences, University of York, Heslington, UK

**Keywords:** Alcohol industry, alcohol research, bias, science, funding, qualitative research

## Abstract

Despite extensive evidence of bias resulting from industry sponsorship of research across health sciences, and longstanding concerns about alcohol industry research funding, there has not been a strong tradition of empirical research on this subject. This study explores researcher decision-making regarding industry funding at the early career stage and the consequences of such funding. Data were derived from semi-structured interviews with researchers working on alcohol policy-relevant topics who first received alcohol industry funding early in their careers (*n* = 7). Data were analyzed thematically using NVivo software. These early-career researchers largely initiated contact with the industry by applying for funding, mostly from industry research funding organizations. Their decisions were shaped by their research environments, where seeking alcohol industry funding early in careers was normative, in large part due to senior colleagues and peers having connections to the industry. Despite being ‘no strings attached’ a ‘snowball’ effect occurred, whereby initial funding led to more industry funding and other opportunities. Receiving early career industry funding had long-term consequences for researchers, not only shaping research networks but also leading to reputational harms as norms around the acceptability of industry funding changed. Exploring this controversial subject in the context of researcher careers adds depth and meaning to larger quantitative studies on bias resulting from industry sponsorship, and identifies mechanisms through which bias may be produced. Further research is required to study the impact of these processes on alcohol policy-relevant research agendas, and also to explore the wider generalizability of these exploratory findings.

## Introduction

Systematic reviews and quantitative studies informed by such approaches have shown that industry-funded research can produce outcomes that support sponsor interests across various subjects (Lesser et al. 2007; Hart et al. 2012; Lundh et al. 2017; Hendlin et al. 2019; Duyx et al. 2020) and move entire research fields toward topics that support commercial, rather than public health interests (Fabbri et al. 2018). Researchers may be to some extent aware of risks of bias posed by industry sponsorship (Fabbri et al. 2018), although there is little research on their decision-making about whether to seek, and experiences of having, such funding, including in relation to otherwise well-studied industries such as tobacco. This has not been seen as a research priority, which could be due in part to the sensitive, often controversial nature of the topic. Further, studies in this area may be challenging to conduct, also due to historic norms on conflict of interest disclosure (Chartres et al. 2019). Qualitative studies that access researchers’ own accounts of the processes by which they came to receive industry funding may be particularly useful in providing foundational data informing thinking about research agendas and how they may be produced.

A series of legal cases in the US against tobacco companies in the 1990s led to the publication of over 70 million pages of internal tobacco industry documents as part of the Master Settlement Agreement (Hurt et al. 2009). This resource enabled researchers to uncover extensive manipulation of science by tobacco industry actors across several decades (Bero 2003, 2005). The tobacco and alcohol industries are connected, for example via co-ownership (Bond et al. 2009; Hawkins and McCambridge 2018), and there have been longstanding concerns about possible bias resulting from alcohol industry funding of research (Babor 2009). These concerns relate to both the individual level, for example, the potential for bias resulting from providing funding to early career researchers (a key tobacco strategy), but also to other levels, with the possibility of cumulative bias on research agendas resulting from industry funding (Babor and Robaina 2013; McCambridge and Mialon 2018). Despite such concerns, there has not been a strong tradition of empirical research on alcohol industry funding of science (McCambridge and Mialon 2018) or on researcher decision-making more broadly.

Recent research has found that both alcohol companies and other alcohol industry-funded groups are extensively involved in funding and/or supporting research, as identified in widely used scientific databases (Golder et al. 2020). One study of the possible bias resulting from alcohol industry funding of research on cardiovascular disease found no evidence of possible funding effects for outcomes other than stroke (McCambridge and Hartwell 2015) and suggested more in-depth research is needed on specific health topics. The $100 million Moderate Alcohol and Cardiovascular Health (MACH) trial received two-thirds of its funding from five global alcohol companies (Rabin 2018). This major study was found to have a biased trial design in a National Institutes of Health review and was terminated in 2018 (Mitchell et al. 2020).

Aside from direct alcohol company funding of research, three grant-making organizations have been prominent in alcohol industry-funded scientific research: the European Research Advisory Board (ERAB), funded by the Brewers of Europe; the Alcoholic Beverage Medical Research Foundation (ABMRF), largely funded by US-based companies; and the *Institut de Recherches Scientifiques sur les Boissons,* which is based in France. The former two closed in 2020 and 2014 respectively, for reasons which are unclear. These research funding organizations are distinct types of social aspect organizations (SAOs) that have largely funded researchers at the early stages of their careers (Babor and Robaina 2013). To our knowledge, there are no dedicated studies of researcher decision-making about, and experience of, these or other sources of industry funding, or the impacts of such funding on subsequent careers.

To address these gaps, we used the findings from a systematic review of concerns in peer-reviewed journals (McCambridge and Mialon 2018) to design an interview study exploring experiences and views on, and decision-making in relation to, alcohol industry involvement in science.

## Methods

We used a qualitative approach for this exploratory study, underpinned by the science and technology studies (STS) literature, where there is an acknowledgment of the role of social interactions in constructing scientific knowledge, and an interest in the values and meanings researchers apply to their scientific work or ‘practice’ (Latour and WooIgar 1986; Latour 1987; Pickering 1992). The present study comprises in-depth interviews with seven researchers who received alcohol industry funding early in their careers. For the purposes of this study, industry funding was defined as research funding from alcohol companies, trade associations, and SAOs, including research funding organizations. Early career included Ph.D. studentship to first project grants or similar awards received as an investigator. This investigation is nested within a larger interview study of alcohol researchers (*n* = 37 participants of 44 invited). Elsewhere, we report on the 16 researchers who developed professional relationships with alcohol industry actors later in their careers (Mitchell and McCambridge 2021), the 14 researchers who had not received industry funding nor had a working relationship with the industry (under review at time of writing), and the views of all 37 researchers on debates on the topic in peer-reviewed journals elsewhere (also under review at time of writing).

Interviews were undertaken between March and July 2019, when the researchers were based in three different countries in North America and Europe; all were mid-late careers at the time of interview. This meant they were reflecting on their early career experiences spanning a 40-year period, approximately 1970–2010. After informed consent was given, semi-structured interviews were carried out by the first author in person, via video, or by telephone. Interviews ranged from 60–105 min in total, with one interview taking place in two parts. We asked the researchers about their careers, their recall of factors informing their decision-making about whether or not to accept industry funding originally, the role of early-career research funding in the development of relationships over time, and other long-term consequences.

Data were analyzed using a form of reflexive thematic analysis (Braun and Clarke 2006; Braun et al. 2019), with each transcript read prior to initial coding using NVivo software by the first author. Coding developed iteratively rather than via a fixed codebook at the start of the process, with the second author reading the transcripts and discussing coding development with the first author. Themes were generated by the first author alongside and subsequent to the coding, and discussions took place between the authors that informed theme refinement and revisions. The first author referred to the literature throughout to make sense of the data (Timmermans and Tavory 2012). The study received ethical approval from the University of York Health Sciences Research Governance Committee. We have removed all identifying information about the participants from the quotes provided below, and do not use pseudonyms to further protect researcher anonymity. Researchers had a variety of consent options to choose from regarding direct quotation, thus we have directly quoted six of the seven researchers included in this paper, with specific permission given to do so.

## Results

Early in research careers, contacts with industry actors were largely initiated by researchers themselves when they applied for funding, often from industry research funding organizations. In some cases, conduits to industry funding requests were already available among colleagues in their training or early career environment. In presenting the findings, we will go on to explore how early career funding led to further contact with industry actors, and shaped subsequent career development.

### Early career research funding

Two main factors informed the original decisions to seek research funding from industry sources. Firstly, the researchers reported issues with the availability of early career research funding. They understood this to be a problem that varied across countries and were generally of the view that it had improved in more recent times. Secondly, applications for industry funding were seen as unremarkable within the researchers’ close academic networks at the time. This took the form of peers submitting applications to the same funder and/or senior colleagues having existing industry connections, including the receipt of funding. Several researchers specifically reported an introduction to an industry funder by their Ph.D. supervisor:

My professor was part of the [SAO] scientific committee at that time and I was introduced by him there.

Senior colleagues also provided some researchers with their first experience of receiving industry funding without requiring them to make a funding application, either by working as part of a team that already received industry funding, or via a discrete industry-funded study on which they were employed. Other environmental influences that encouraged decisions to seek funding in the formative years also operated through their broader networks. This included interpersonally, via other early career researchers doing similar work at other universities, for example, and institutionally, when information about industry funding opportunities was disseminated by their own university.

Once researchers became aware that industry funding was available and that colleagues had successfully secured such grants, they reported seeking funding themselves, often from the same organization. Particularly in earlier decades, researchers received initial industry funding from trade associations, either via advertised open calls or through existing funding senior colleagues had sought and secured. Researchers so funded reported that they did not experience any interference from trade associations in the research process, and levels of contact during the conduct of the study varied, ranging from colleagues having regular face-to-face meetings to update the funder, to having no contact at all.

In more recent decades, industry research funding organizations such as ABMRF and ERAB were more prominent, with both organizations advertising open funding calls. One researcher based in North America reported that it was normative to apply for ABMRF funding at the early career stage, noting that a broad range of now prominent researchers had received such funding:

A lot of the current leaders in alcohol research…were funded by ABMRF grants.

Early career researchers based in Europe submitted applications to open funding calls for small-scale studies, or for travel grants to conferences they would have otherwise been unable to attend:

So the first bit of funding I got from [industry research funding organisation] was a travel grant actually. So there wasn’t a lot of funding at [university name] for going to conferences…I wouldn’t have been able to present that work without that money because there was no other funding [available].

Thus, industry funding complemented support from other sources, with funding acquired from a range of industry and non-industry sources. This was particularly true of the early development of new research ideas or directions:

It was too early to get any funding from the larger research councils, so we were able to get this smaller funding for this [from industry research funding organisation]. And then from that we applied for some [funding from public body].

In other cases, researchers had intervention-related or extra materials for a study from an industry source in addition to the main funding coming from other sources. Some researchers reflected that early-career industry funding played a crucial role in career development:

If I hadn’t got that grant, my career would have looked very different…the work that I went on to do with [non-industry funder], developing a range of things, came directly from that grant.

Industry research funding organizations paid the direct costs of the research only, with researchers reporting that the amounts of money available in industry research grants were relatively small, particularly in comparison to publicly-funded studies. At times, researchers received less funding than requested, leaving researchers to manage university concerns about meeting the direct costs of the study, as well as the lack of overheads. Most reported that contacts with funders were minimal and usually took place via e-mail or other correspondence, and that industry research funding organizations did not in any way attempt to influence the conduct of the research after the award of funding. Indeed, all interviewees emphasized that they experienced no interference in grant-related decision-making by industry funders once awarded:

We never had money from the industry that ha[d] strings attached.

### The ‘snowball effect’: subsequent researcher-industry relationships

Whereas first contacts between industry and researchers were largely researcher-initiated, within an environment which encouraged this activity, subsequent relationships and involvement in industry-related networks were developed by both parties. All seven researchers went on to receive grants from non-industry sources, such as governments and charities. Researchers reported gradually building their networks as they developed their careers and knowing which organizations other researchers were receiving funding from, particularly those at similar career stages, was part of this. This information was taken into account in subsequent considerations of applying for industry funding where areas of interest converged:

So [colleague] approached me and said, ah I see you’ve got money from [industry research funding organisation]; I’m interested in doing a similar study.

Some of these researchers also received funding from SAOs after initial awards by industry research funding organizations. These grants were not made through open calls but were described as part of the evolving relationships between these organizations and researchers. Publication opportunities arose in similar ways. Research funding could also be used to later involve other researchers, thus building networks in ways that may not have been possible with other funders. As one researcher recalled:

[A colleague] basically said, look, we’re going to get this money. I was thinking, right, okay, because with most funders that’s not how things work.

Following the development of these networks, five of the seven researchers reported being invited, and all agreed, to perform expert advisory roles for industry bodies. Researchers were often invited to perform these roles by colleagues, including those who had moved from employment with a public body to an industry organization. For industry research funding organizations, this typically comprised membership of a scientific committee that made decisions about the allocation of funding, and researchers also described undertaking peer review activities. Scientific committee members could also be involved in inviting colleagues to perform reviews of applications under consideration. The majority of researchers reported no industry interference when performing such activities:

It’s an entirely independent process, the research bids come in, they go out for peer review, like anything else would, they get selected and funding is based on those reviews and equality.

On the rare occasions where industry research funding organizations attempted to interfere in scientific committee decision-making processes, researchers reported refusing such requests. Membership of scientific committees for research funding organizations usually lasted several years, and the role could be paid or unpaid, often requiring significant time commitments from the researchers. Researchers recalled face-to-face contact with industry research funding organization employees when sitting on these committees, and that contact with the alcohol company or trade association funders of those groups was limited. Recruitment to expert advisory roles for SAOs was reported to operate somewhat differently than for research funding organizations, with researchers reporting being invited directly by SAO employees, as well as by colleagues with an existing relationship with the SAO. These kinds of roles and activities followed on from receipt of early career funding in most cases, and thereafter were mutually reinforcing as researchers became better known in these circles:

We were in contact with [SAO] because we knew each other and also, more recently, I was part of the scientific committee, so I just said I have an idea [on a specific topic]… and we wrote a proposal. And if they were interested they funded [the study].

Several researchers reflected that the aims and activities of industry research funding organizations seemed to change over time, and some questioned whether SAOs really sought to achieve their stated goals. In both cases, these researchers perceived this to be linked to evolving practices in corporate social responsibility that led to inappropriate requests to bypass scientific norms.

Following receipt of early career industry funding, five researchers reported an invitation to at least one industry-funded event by industry research funding organizations, by SAOs, and/or by major alcohol companies, which all five accepted. It was common to have direct contact with global alcohol producer employees at these events. Researcher expenses were paid, with one researcher reporting that this helped attract researchers:

I saw a lot of these perks [event expenses and nice locations] not so much as attempts to influence the scientists, or to do anything illegitimate, but it was a way of attracting [researchers].

The other two researchers reported being invited to and attending, smaller meetings with an SAO, in one case to discuss their existing involvement in an SAO-funded project, and in another to share their research findings and discuss possible industry funding, which they subsequently received. SAO employees also reached out directly to researchers when they attended non-industry funded events:

I was at an event where I was doing a talk about [industry-funded study] and the…[SAO senior employee] was there, and she said…it would be interesting if you would be able to come and talk to our people about what you found out. We spoke to them about what they were doing and what the impact was, and they were interested in doing some more research… Out of those negotiations [the SAO part-funded a small research project].

Cold calling, where there had been no prior contact with the organization, was rare.

Early career access to industry funding, subsequent performance of expert advisory roles and other scientific activities, and attendance at both industry-funded and non-industry-funded events and other meetings provided opportunities for the development and maintenance of industry-researcher networks.

### Potential adverse consequences of early career industry funding

It was common for these researchers to report damage to their reputation as a result of receiving industry funding and/or performing expert advisory roles and/or other industry-linked activities. Most researchers were not aware of the potential long-term consequences of accepting industry funding at the early career stage, reporting that broader debates about alcohol industry involvement in science had not entered mainstream discussions at that time. This changed later in their careers when this subject became more controversial and norms regarding industry funding changed:

I am aware that there are colleagues that would think differently of my work as a result of my industry connections. But I’ve had [industry connections] since I started alcohol research and I can't go back and change my CV, so…there seems to be very little more to be gained in terms of disadvantage by continuing to do something that I think is a really important piece of work.

Impacts on reputations could entail further adverse consequences, for example with researchers reporting losing opportunities and/or conflicts as a result of their receipt of industry funding. All reported negative perceptions of industry connections:

You worry all the time that somebody is going to come up to you and tell you, oh but that’s rubbish because you got it funded by [industry research funding organisation], and you feel terrible.

The researchers had various ambivalences about their experiences with industry research funding organizations and other industry groups. Some had decided that they would no longer accept industry funding or perform expert advisory roles. Some recognized that perceptions of industry funding had changed in recent years, and better appreciated now that there were deleterious impacts on research agendas. For some, this was balanced with the view that industry funding facilitated career development and advances in research. Decision-making was characterized by conflicts of these kinds, and these researchers reported benefitting little from institutional guidance on these matters and were left to navigate these complex decisions for themselves:

People are probably working it out for themselves, having to work it out for themselves.

## Discussion

Insights from this exploratory study have enabled us to identify the crucial role of norms and socialization processes in shaping decision-making about industry funding. This is, however, a small sample, drawn from three countries and covering approximately four decades. The interviewees first accepted industry funding at a time when it was more acceptable, whereas they were reflecting in the interviews at a time when it is now considered more questionable. Thus, retrospective accounts may be influenced by changing norms and/or other individual motivations. Further, the second author has played a role in debates about industry funding within the research community (for example, Andréasson and McCambridge 2017). Although the response rate suggests that the team was trusted to undertake this study, it remains possible that this may have influenced the content of the interview data. All interviews were undertaken by the first author, providing mitigation of this risk, and we were impressed by the willingness of the researchers to engage with the process and share what were, for some, difficult personal experiences in the interviews. In the analysis, we were led by the data in our generation of themes, and this process was shaped by the earlier systematic review (McCambridge and Mialon 2018) as it has informed the design of the interview guide and the study more broadly. As with any such study, this must be acknowledged as constituting a potential limitation in ways that may not be apparent to the authors. Accordingly, we have sought to present our findings in ways that allow the reader to make this kind of assessment.

Our study firmly points to the importance of research environment as context, and to the intrinsically social processes at work therein, that shape early career decision-making regarding industry. These researchers largely initiated contact with industry by applying for funding early in their careers, when applying for such funding was normative, in no small part due to senior colleagues and peers having connections to industry. Attention to individual decision-making about working with industry must therefore take account of these broader contexts (Adams 2016). Replicating, deepening, and extending the findings of the present study offers a clear direction for further research, and we provide a set of possible questions for future study in Box 1. Addressing the questions identified here will help develop understanding of how these issues play out in the various disciplines engaged in the alcohol field, where scientific norms and values are shaped by patterns of interactions that may be more or less amenable to building relationships with industry. The questions have been framed in ways which invite their application to other fields of research and should be studied across low, middle, and high-income countries, where the availability of non-industry funding sources varies (Martin et al. 2016).

Box 1. Questions for further research

- What role does the research environment, including factors such as mentor guidance, events attended, and committee work, play in shaping early career decision-making regarding industry funding?
- How do early career industry funding grants impact on subsequent researcher career trajectories, including research agendas and beneficial and/or adverse consequences?
- Are there snowball effects leading to altered network memberships, and if so, to what extent are industry actors key nodes in, or influences on, these networks?
- How do environmental and individual-level factors interact to produce snowball effects, and where are they most likely to arise?
- How does industry involvement in science take advantage of limited public funding, and how successful are the various strategies used?
- To what extent do scientific norms and values regarding industry differ between the various disciplines engaged in alcohol and other interdisciplinary forms of research?

Despite being ‘no strings attached’, the snowball effect entailed early career funding leading on to further industry funding, engagement in industry associated networks, and shaping career opportunities in other ways. Receiving early career industry funding thus had long-term consequences for these researchers, some of which were adverse. These processes are subtle, and a key strength of this study is that a coherent understanding over time becomes possible when adopting the approach of examining these processes within career contexts. The snowball effect is probably made more likely by the precarious nature of scientific work (Lave et al. 2010), suggesting that the present findings may have wide generalizability. It is important, however, to emphasize that the snowball effect identified here requires further study. The seven researchers worked in social sciences and/or health, therefore it may be useful to build on these findings by identifying to what extent distinct research ‘bubbles’ or epistemic cultures (Knorr-Cetina 1999) exist within the broader territories of alcohol and addiction research.

This study explores contemporary reflections on early career experiences, thus it is imperative to identify to what extent the exploratory findings apply to early career researchers today. For example, although ABMRF and ERAB have been dissolved, there remains a need to address existing concerns (Babor 2009; Babor and Robaina 2013; McCambridge and Mialon 2018) about the long-term impacts of these organizations’ activity on both knowledge on alcohol and the alcohol research agenda. That researchers reported various forms of subversion of research funding norms highlights the need to study SAO involvement in science in particular, although this may be expected to be challenging to study.

More broadly, our qualitative data add meaning and depth to quantitative studies on bias resulting from industry sponsorship of research, including car manufacturers and pharmaceutical, tobacco, and food and beverage industries (Lesser et al. 2007; Hart et al. 2012; Lundh et al. 2017; Hendlin et al. 2019; Duyx et al. 2020). Such studies raise the specter of funder interference; our findings suggest that even where funder interference does not occur in the conduct of the funded study, a small industry investment at the early career stage can have long-lasting impacts on subsequent research and career development. The key contribution of this study is that it advances understanding of precisely *how* these processes may unfold. Further research is required on early career funding from industry sources, even when it appears to have no strings attached, and the extent to which applying for, and receiving, industry funding may shape individual careers and research agendas in unforeseen ways for years to come.
